# Editorial: Advances in radiation research and applications: biology, environment and medicine

**DOI:** 10.3389/fpubh.2026.1853477

**Published:** 2026-04-29

**Authors:** Yi Xie, Fei Tuo, Ning Cao, Chang Xu, Bing Wang

**Affiliations:** 1 Department of Medical Physics, Institute of Modern Physics, Chinese Academy of Sciences, Lanzhou, China; 2 National Institute for Radiological Protection, Chinese Center for Disease Control and Prevention, Beijing, China; 3 Department of Radiation Oncology, University of Washington School of Medicine, Seattle, WA, United States; 4 Institute of Radiation Medicine, Chinese Academy of Medical Sciences and Peking Union Medical College, Tianjin, China; 5 Institute for Radiological Science (NIRS), National Institutes for Quantum Science and Technology (QST), Chiba, Japan

**Keywords:** radiation biology, radiotherapy, environmental radiation, radiation epidemiology, public health, low-dose radiation, radiation risk

Radiation remains a cornerstone of modern society, playing indispensable roles in medicine, industry, agriculture, and scientific research, while simultaneously posing complex challenges to human health and public safety. The Research Topic “*Advances in radiation research and applications: biology, environment and medicine*,” published in Frontiers in Public Health brings together 21 contributions that reflect the rapid evolution and growing interdisciplinarity of contemporary radiation research. Collectively, these articles span fundamental biology, clinical and translational medicine, environmental exposure, and population-level public health perspectives.

A unifying theme across this Research Topic is the reconceptualization of radiation- not simply as a therapeutic tool or environmental hazard, but as a multifaceted biological and societal factor that influences health across molecular, individual, and population scales. By integrating mechanistic insights with clinical innovation and epidemiological evidence, these contributions highlight the increasingly systems-level nature of contemporary radiation research.

## Overall structure and article types

The 21 articles included in this Research Topic can be broadly categorized into four types. Original Research Articles (15 studies) form the core of the Research Topic, encompassing experimental mechanistic investigation, clinical modeling approaches, epidemiological analyses, and technological innovations in radiation monitoring and detection. Review Articles (four studies) synthesize current knowledge on topics such as radioresistance, metabolic reprogramming, radiation exposure risks, and the abscopal effect, offering integrative perspectives on rapidly evolving areas of radiation research. In addition, one Study Protocol presents a prospective cohort design aimed at advancing understanding of the long-term health effects associated with low-dose radiation exposure, while a Commentary provides methodological critique and guidance for epidemiology and risk assessment research.

This distribution underscores the Research Topic's strong emphasis on data-driven discovery, while maintaining space for conceptual synthesis, critical reflection and future-oriented research design.

Geographically, the contributions originate predominantly from China, with additional contributions from Germany and the United Kingdom, reflecting both regional strengths in radiation research and growing international collaboration. Collectively, the articles may be broadly divided into experimental investigations focused on biological mechanisms and clinical, environmental and epidemiological studies addressing human health risks and translational applications.

## Thematic integration across disciplines

Despite their diversity, the contributions to this Research Topic can be conceptually organized into three interconnected domains.

### Radiation biology and mechanistic insights

Several studies focus on elucidating the molecular and cellular mechanisms underlying radiation effects. Investigations using proton and carbon ion irradiation reveal processes such as immunogenic cell death and ferroptosis as critical links between radiation and tumor control. Other studies highlight the role of key molecular mediators including HMGB1, PCBP1, and TRAF2, in connecting radiation exposure to immune signaling, oxidative stress, inflammation and fibrosis. Beyond ionizing radiation, work examining the metabolic and reproductive effects of electromagnetic fields further broadens the biological scope of the Research Topic.

Together, these studies emphasize oxidative stress, immune modulation and metabolic reprogramming as central nodes in radiation response, providing a mechanistic foundation for downstream translational and public health research.

### Clinical translation and precision medicine

A second cluster of articles centers on improving patient outcomes through predictive modeling, advanced imaging and novel therapeutic strategies. Multiple studies develop nomograms and radiomics-based models to predict radiation-induced injuries, such as enteritis and vaginal injury, or to enhance diagnostic performance in breast nodule assessment. Imaging-based approaches, particularly magnetic resonance imaging, demonstrate promise as non-invasive tools for early detection and monitoring of radiation damage.

In parallel, innovative therapeutic interventions, including adipose-derived stem cell exosome-based approaches, illustrate emerging strategies for mitigating radiation-induced tissue injury.

These contributions collectively highlight a shift toward precision radiation medicine, in which individualized risk stratification and targeted interventions are informed by quantitative models and imaging biomarkers.

### Environmental and public health perspectives

The third thematic domain addresses radiation exposure beyond the clinical setting, emphasizing occupational, environmental, and population-level health effects. Large-scale epidemiological studies explore associations between chronic radiation exposure and adverse outcomes such as sleep disorders, dyslipidemia and multi system morbidity. Survey-based studies reveal limited radiation literacy and elevated anxiety among both residents and healthcare workers, underscoring the importance of effective risk communication and education.

Complementing these findings, review articles and study protocols stress the significance of cumulative exposure, low-dose risk assessment and long-term cohort studies. Collectively, these contributions reinforce radiation's relevance as a public health concern and highlight the need for evidence-based policies, protection strategies, and emergency preparedness, particularly in the context of radiation accidents and environmental contamination.

### Methodological insight and scholarly dialogue

A distinctive feature of this Research Topic is explicit engagement with methodological rigor and scholarly exchange, exemplified by the inclusion of both a study protocol and an accompanying critical commentary.

The protocol article proposes a prospective cohort study of occupational workers exposed to low-dose radiation in Chongqing, with the aim of evaluating exposure–lag relationships and long-term health effects using advanced statistical modeling approaches. By outlining study design, exposure assessment strategies, and planned analytical frameworks in advance, this contribution reflects growing recognition of the importance of transparency and reproducibility in radiation epidemiology.

Importantly, the protocol is directly followed by a general commentary that critically examines key aspects of the proposed study design. This commentary highlights potential challenges related to confounding control, sample size justification, feasibility and statistical power, and provides constructive recommendations aligned with international best practices in radiation risk assessment. Together, this scholarly dialogue underscores that methodological scrutiny is not ancillary but integral to advancing robust and credible evidence, particularly in the context of low-dose radiation research, where uncertainties are substantial and effect sizes are often modest.

Together, the protocol and commentary illustrate how open academic debate can enhance study quality, refine research questions, and ultimately strengthen the evidence base used to inform public health policy and radiation protection standards. Their inclusion in this Research Topic emphasizes our commitment not only to scientific discovery, but also to methodological excellence, critical reflection, and continuous improvement in radiation research.

## Logical connections across the 21 articles

Despite their varied methodologies and focal points, the articles in this Research Topic form a coherent continuum. Mechanistic studies provide the biological basis for understanding radiation effects; translational and clinical studies build upon these insights to improve diagnosis, prediction and therapeutic intervention; epidemiological and public health investigations extend these findings to real-world populations and long-term outcomes; and reviews and commentary integrate existing knowledge while refining methodological frameworks.

This bench-to-bedside-to-population trajectory illustrates how fundamental discoveries in radiation biology can ultimately inform clinical practice, public health strategies and policy development.

## Big picture and significance

The overarching contribution of this Research Topic lies in its demonstration that radiation research is entering a systems-level era. Key emerging concepts include radiation as a biological modulator influencing immunity, metabolism, and cell fate decisions; the integration of multi-scale data from molecular pathways to population cohorts; the advancement of precision and personalization enabled by predictive modeling and advanced imaging, and the growing recognition of radiation's public health and societal implications, particularly in the context of chronic low-dose exposure. At the same time, the Research Topic highlights unresolved challenges, including uncertainties in low-dose risk assessment, inter-individual variability in radiation susceptibility, and persistent gaps in radiation literacy among the public and health professionals.

## Conclusion

This Research Topic provides a comprehensive and forward-looking overview of contemporary radiation research across biology, medicine, and the environment. By bridging mechanistic insights with clinical innovation and public health relevance, it underscores the necessity of interdisciplinary collaboration in addressing both the benefits and risks of radiation exposure. Collectively, the 21 contributions advance our understanding of radiation as both a powerful therapeutic modality and a complex environmental factor, offering valuable guidance for future research, clinical practice, and policy development.

